# Microbiologic characterisation of bacterial infections in children with atopic dermatitis

**DOI:** 10.4102/sajid.v37i1.368

**Published:** 2022-03-31

**Authors:** Nkosinathi O. Zwane, Josiah T. Masuka, Antoinette V. Chateau, Anisa Mosam

**Affiliations:** 1 Department of Dermatology, Faculty of Medicine, University of KwaZulu-Natal, Durban, South Africa

**Keywords:** atopic dermatitis, children, bacterial infection, Staphylococcus, Streptococcus, antibiotics

## Abstract

**Background:**

Patients with atopic dermatitis (AD), the commonest chronic inflammatory skin disease are often colonised and infected by *Staphylococcus aureus*. In this study, we aimed to determine the type and antibacterial sensitivities of the bacteria infecting eczematous lesions in children with AD and to recommend first-line antibiotic therapy.

**Methods:**

A prospective study was conducted from June 2020 to June 2021 in children with AD presenting with a cutaneous infection at the King Edward hospital VIII outpatient dermatology clinic. Swabs were collected for microbial culture, confirming infections and assessing antibiotic sensitivity for infected sites.

**Results:**

Ninety six children were recruited during the study period with a mean age of 4.3 ± 3.4 years. The commonest cause of bacterial infection was *Staphylococcus aureus* seen in 74 (77.1%) cases, followed by *Staphylococcus aureus* and *Group A* β*-haemolytic streptococcus (GAS)* co-infection in 22 (22.9%) cases. The majority of these infections were observed on the lower limbs in 50 (52.08%) cases and in moderate 37 (38.5%) cases and severe eczema cases of 38 (39.6%) in AD. There was no gender predilection. *Staphylococcus aureus* was sensitive to amoxicillin-clavulanic acid in 57 (77.0%) cases, cloxacillin in 53 (71.6%) cases and clindamycin in 24 (32.4%) cases, whereas *GAS* was mostly sensitive to ampicillin in 10 (45.5%) cases. No swabs retained a resistant strain.

**Conclusion:**

*Staphylococcus aureus* is the commonest bacterial cause of cutaneous infection in children with AD in our setting. Amoxicillin-clavulanic acid and cloxacillin remain the most sensitive therapeutic options for this infection, however, a larger study is required to explore resistance strains, if any, in our setting.

## Introduction

Atopic dermatitis (AD) is the commonest chronic, allergic inflammatory disease with a prevalence of 9.9% in the primary care population.^1^ A higher prevalence in the range of 10.0% to 25.0% is observed in children within industrialised nations.^1^ Atopic dermatitis contributes to a considerable public health burden as it is associated with increased direct and indirect healthcare costs.^2^ Furthermore, AD is often complicated by recurrent bacterial and viral infections which can lead to significant complications when left untreated.^3^ The infectious complications of AD include skin and soft tissue infections, eczema herpeticum, eczema vaccinatum, bacteraemia, endocarditis, osteomyelitis and septic arthritis.^4,5,6^

Patients with AD are at increased risk of cutaneous and subsequent systemic infection because of their aberrant cutaneous innate and adaptive immune systems.^4,7^ The reduction of antimicrobial peptides such as cathelicidins, increased skin pH and Th2 cytokines – such as interleukin 4 and 13 and the dysbiotic skin flora contribute to this susceptibility.^3^ Chronic scratching of pruritic eczematous skin leads to worsening of an existing defect in the epidermal barrier, allowing the invasion of bacteria into the skin and bloodstream.^5^ Furthermore, colonisation with *Staphylococcus aureus* is common in patients with AD.^4^ Predictably, *S. aureus* is the commonest cause of bacterial skin infections in these patients because of its ability to evade cutaneous defenses.^4,7^ The second most common cause of cutaneous infections in AD is *Group A* β*-haemolytic streptococci (GAS)*.^4,8^ Eczematous lesions in flexural areas, higher grades of itchiness and atopy have previously been associated with an increased risk of bacterial infection.^9^ On the other hand, antibiotics and bleach baths have been used to decolonise and thus reduce the risk of bacterial infection in AD patients.^4,10^ However, there is insufficient evidence and controversy, especially with the use of antibiotics in this setting.^4^ Bleach baths may improve the symptoms of AD by eradicating bacteria, especially *S. aureus.*^10^ They seem to be safe on human skin and do not appear to disrupt the epidermal barrier.^10^

Given that no studies have evaluated bacterial infections observed in AD in South Africa, we set out to determine the type and antibacterial sensitivities of the bacteria causing infections in children with AD in our setting. In addition, we describe the sites of infection, AD severity and the extent of bleach bath use in these patients. Studying the bacterial community infecting eczematous plaques may offer valuable information for formulating effective antibiotic therapies and for predicting AD outcomes.

## Methods

### Study setting

Between June 2020 and June 2021, all children with atopic eczema attending the dermatology clinic at King Edward hospital, a tertiary hospital in Durban, KwaZulu-Natal, South Africa were screened for inclusion in our study. All children between the ages of 6 months and 12 years with clinically suspected infected eczema were included in the study after assent or consent was signed by them and their parents. Atopic dermatitis was defined as the UK working criteria, that is, pruritus associated with three of the following: visible or history of flexural dermatitis, history of xerotic skin in the past year, history of atopy and/or onset under two years of age.^11^ Infected eczema was defined as a case presenting with pustules, purulent discharge, weeping and/or crusting.^8^

### Data and sample collection

Socio-demographic, clinical and microbial data were collected onto a patient record form and later transferred onto a Microsoft Office Excel^TM^ spreadsheet (Microsoft Corporation, Redmond, WA, United States [US]). The following information was collected and/or calculated – age, gender, scoring for Atopic Dermatitis (SCORAD),^12^ site of infection, causative organism and its bacterial sensitivity. The infected area was cleaned with sterile normal saline solution and then swabbed. Swabs were placed into sterile, screw-cap containers with Aimes transport media and sent to the National Health Laboratory Service (NHLS) for plating, culture and bacterial sensitivity testing.

### Statistical analysis

Descriptive statistical analysis was done using the Statistical Package for Social Sciences (SPSS) version 16, (IBM Corporation, Somers, NY, US). Frequencies and percentages were used to summarise categorical variables. Convenience sampling was used with all consenting or assenting children with infected AD included in the study.

### Ethical considerations

Assent or consent was obtained from the children and their parents or guardians as appropriate. Ethical approval for the study was obtained from the hospital and the University of KwaZulu-Natal Biomedical Research Ethics Committee, BREC reference: BE417/18.

## Results

**FIGURE 1:**
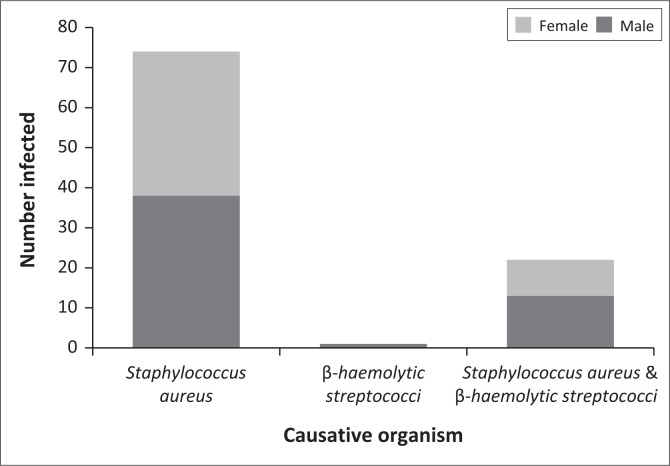
Causative organisms of secondary infection.

A total of 96 participants were recruited for the study. The mean age of the participants was 4.30 ± 3.35 years with a slight predominance of males 52 (54.1%) and females 44 (45.8%). Most of the infections were observed in patients as moderate 37 (38.5%) and severe eczema 38 (39.6%) on the SCORAD severity score highlighted in Table 1. The most affected sites were the lower limbs in 50 (52.1%) patients with the remainder of the infections observed on the face, neck and upper limbs. *Staphylococcus aureus* caused 74 (77.1%) cases of infections including 22 (22.9%) co-infections with *GAS*, whereas *GAS* only had one case of infection recorded as shown in Figure 1. *Staphylococcus aureus* was sensitive to amoxicillin-clavulanic acid in 57 (77.1%) cases, cloxacillin in 53 (71.6%) cases and clindamycin in 24 (32.4%) cases, whereas *GAS* was mostly sensitive to ampicillin in 10 (45.5%) cases. Forty two of the 95 isolates of *S. aureus*, were not sensitive to cloxacillin as shown in Table 2. Of these 42, only 14 (33.3%) were sensitive to amoxicillin-clavulanic acid. None of them were sensitive to amoxicillin, ampicillin, clindamycin, azithromycin, erythromycin or clindamycin. Only one participant amongst the study participants reported the use of antiseptic/bleach baths.

**TABLE 1:** Patient demographic characteristics.

Characteristic	Male	Female	Overall
**Age/years**			
Mean ± s.d.	4.25 ± 3.33	4.35 ± 3.37	4.30 ± 3.35
Median	3	3	3
IQR	1.87–6.00	1.48–6.75	1.67–6.00
**Duration with eczema/years**			
Mean ± s.d.	3.25 ± 3.30	3.49 ± 3.01	3.36 ± 3.18
Median	2	2	2
IQR	0.5–5.0	1.0–5.0	1.0–5.0
**Infected body site**			
Face	2	4	6
Neck	5	6	11
Upper limbs	1	0	1
Lower limbs	25	25	50
Other sites	2	2	4
**SCORAD**			
Mild	10	9	19
Moderate	16	21	37
Severe	17	21	38
Previous infection	13	13	26

**Total**	**52**	**44**	**96**

s.d., standard deviation; IQR, interquartile range; SCORAD, Scoring for atopic dermatitis.

**TABLE 2:** Antibiotic susceptibility pattern of isolated bacteria from skin swab culture.

Isolate (*N*)	Amoxy		Ampic		Amoxclv		Cloxa		Clinda		Azithro		Erythro		Vanco	

	*n*/*N*	%	*n*/*N*	%	*n*/*N*	%	*n*/*N*	%	*n*/*N*	%	*n*/*N*	%	*n*/*N*	%	*n*/*N*	%
*Staphylococcus aureus* (74)(S)	0	-	1	1.4	57	77.0	53	71.6	24	32.4	12	16.2	13	17.6	0	-
*GAS* (22)(S)	2	9.1	10	45.5	5	22.7	1	4.5	6	27.3	3	13.6	3	13.6	0	-
Total (96)(S)	2	2.1	11	11.5	62	64.6	54	56.3	30	31.3	15	15.6	16	16.7	0	-

Amoxy, amoxicillin; Ampic, ampicillin; Amoxclv, amoxicillin-clavulanic acid; Cloxa, cloxacillin; Clinda, clindamycin; Azithro, azithromycin; Erythro, erythromycin; Vanco, vancomycin; S, sensitive; *GAS, Group A β-haemolytic streptococci*.

## Discussion

In this study, we explored the type and antibacterial sensitivities of the bacteria causing cutaneous infections in children with AD managed at a tertiary dermatology clinic in KwaZulu-Natal, South Africa. Largely, this population did not use antiseptic baths for the prevention of cutaneous super-infections.^10^ *Staphylococcus aureus and GAS* co-infection are the commonest observed secondary cutaneous infections. Unlike other studies, no enterococci or other bacteria were cultured in our study.^13^ *Staphylococcus aureus* was mostly sensitive to amoxicillin-clavulanic acid and cloxacillin whereas *GAS* was mostly sensitive to ampicillin.

As has been documented in other studies, staphylococcal infection was the predominant cutaneous bacterial infection in our setting.^7,8,13^ However, concomitant *GAS* infection should always be borne in mind as previous studies indicate rates as high as 62.0%.^7,8^ The latter validates the empiric use of amoxicillin-clavulanic acid and flucloxacillin in the treatment of infected AD patients as both these medicines are almost always effective for both infections.^8^ The 23.0% amoxicillin-clavulanic acid non-sensitive *S. aureus* isolates potentially indicate methicillin-resistant *Staphylococcus aureus* (MRSA). In other settings, MRSA has been noted in a range from 6.4% to 30.5%.^13^ In some of these settings, erythromycin, clindamycin and cephalosporins have been recommended for the treatment of MRSA.^14,15^ However, in our setting, clindamycin and erythromycin are not effective options,^13^ given the 67.6% and 83.4% resistance levels, respectively. Consultation with infectious diseases and medical microbiology specialists will be needed in MRSA cases. Furthermore, regular antibiotic sensitivity testing and therapeutic drug meetings should be undertaken to re-evaluate our therapeutic armamentarium. This is especially important in patients with AD as skin infections are more common in those with severe AD.^13^

In keeping with our findings, infections rarely occur on the face or abdomen,^9^ but it is not clear why the lower limbs had more infections in our study. Increased exposure of the limbs to the external environment may explain this finding. Previous studies indicate that infections occur more in flexural areas compared to extensor surfaces, although in patients of African descent, the extensor surfaces may be favoured.^7,9^ Most of the infections were observed in AD patients with moderate or severe AD, suggesting a relationship with AD severity as previously reported by Samosir et al.^13^ We observed a slight male predominance, unlike in the study by Samosir et al. who reported more infections in girls compared to boys.^13^

The major strength of this study is the characterisation of the bacteria commonly responsible for cutaneous infections in our AD patients. Together with the observed antibiotic susceptibilities, this study provides evidence in support of the continued empiric use of flucloxacillin for infected lesions in patients with AD in our setting. Thus, promoting the rational prescription of cost-effective, readily available and easily accessible narrow-spectrum antibiotics.^16^ However, our study had several limitations. Firstly, the criteria for AD diagnosis we used can potentially miss infected AD cases presenting without clinical indication. Secondly, our sample was too small to fully explore streptococcal infections and their antibiotic sensitivities given that they are the second commonest bacterial infection observed in AD. Thirdly, we did not include non-infected patients or collect data on previous antibiotic exposure, HIV status or explore nasal bacterial carriage in our patients and their family members. These variables would have assisted in comparative analyses to explain some of our findings. We recommend a larger study across all age groups to explore the prevalence of bacterial infections in AD patients further exploring the parameters we observed and those missed with our current study.

## Conclusion

*Staphylococcus aureus* is the commonest bacteria causing cutaneous infections in children with AD with lower limbs being the commonest site of infection. Children with moderate-to-severe AD were most susceptible. Amoxicillin-clavulanic acid and cloxacillin remain the most sensitive empiric therapeutic option in this setting. However, pus swab guided therapy may be required in patients unresponsive to any of these medications because of concomitant *S. aureus/GAS* co-infection, where the latter is more susceptible to ampicillin. Clinicians should only use antibiotics when necessary and limit the use of broad spectrum antibiotics in these patients to aid antibiotic stewardship efforts.^17^
